# Editorial: Mental illnesses within correctional populations

**DOI:** 10.3389/fpsyt.2023.1145921

**Published:** 2023-02-20

**Authors:** Emily D. Gottfried, Michael J. Vitacco, Mini Mamak

**Affiliations:** ^1^Community and Public Safety Psychiatry Division, Medical University of South Carolina, Charleston, SC, United States; ^2^Department of Psychiatry and Health Behavior, Institute of Public and Preventive Health, Augusta University, Augusta, GA, United States; ^3^Forensic Psychiatry Program, St. Joseph's Healthcare Hamilton, Hamilton, ON, Canada; ^4^Department of Psychiatry and Behavioural Neurosciences, McMaster University, Hamilton, ON, Canada

**Keywords:** correctional population, forensic treatment, mental illness, juvenile offenders, risk assessment

Individuals serving time in correctional facilities, including adults and youths of both sexes, have a higher prevalence of mental illness compared with the general population (1–3). Several factors contribute to mental illness in this population, including a higher level of adverse childhood experiences (4, 5); these individuals not receiving appropriate level or quality of community-based treatment (6); treatment not directly targeting criminogenic needs or mental health concerns (7); and with strained institutional budgets and inadequate resources, many incarcerated persons do not receive sufficient or appropriate treatment (8). The barriers inherent to incarceration prevent both the adequate treatment of mental illness and the resolution of factors that contribute to recidivism (9).

In this Research Topic, the authors assessed the impact of legal traditions on mental health treatment, differences between youths who desisted from serious violent offending from those who did not, risk factor differences for future sexual offending based on IQ scores, and auditory emotion processing in a forensic and non-forensic group of men with schizophrenia.

Beis et al. provided an update to Crocker et al.'s study (10) on forensic mental health systems across the world. Like Crocker et al. (10), the authors examined international legal frameworks to include Common, Civil, and Islamic Law. They described the location of forensic services, number of forensic beds, and total number of psychiatric care beds in select countries in Europe, the Americas, Asia, Africa, and Oceania. In Europe, England, and Wales were reported to have the greatest number of forensic beds (4,250 high/medium secure combined), while Austria was reported to have the least (384). In the Americas, the United States was reported to have the greatest number of forensic beds (7,835), while Chile was reported to have the least (209). In Asia, Russia was reported to have the greatest number of forensic beds (12,022 high/medium secure combined), while Pakistan was reported to have the least (33). In Africa, South Africa was reported to have the greatest number of forensic beds (1,676), while Nigeria was reported to have the least (22). Australia was reported to have 680 forensic beds and New Zealand was reported to have 221. The authors concluded that there was a “need for the optimization of forensic treatment standards on an international level.”

Barra et al. followed 129 young (i.e., 14–23 years old) men who were incarcerated in a juvenile detention facility for up to 15 years to assess factors relevant to serious, violent, and chronic (SVC) offending. The authors reported a high prevalence of mental health symptoms in the sample, especially externalizing problems. Encouragingly, the results indicated that some of the youths were able to desist from SVC offending, even those with a history of severe offending and highlighted “the need of effective early identification to reduce young people's risk of engaging in serious, violent, chronic delinquent careers.” Those who desisted from SVC offending had fewer psychiatric symptoms, including fewer externalizing behaviors, attentional symptoms, serious alcohol use, and personality pathology. “Alarming alcohol use” was shown to significantly contribute to future SVC offending. The authors recommended that youths at risk for future serious offenses would benefit from mental health treatment focused on problematic alcohol use.

Vicenzutto et al. noted important differences in the “clinical and criminological origins” in men who had committed sexual offenses based on IQ scores. The authors assessed 137 men who had been adjudicated not criminally responsible for a sexual offenses and the role of IQ scores on static and dynamic risk factors. Compared to men with higher IQ scores, those with low IQ (i.e., 70 or less) were younger; more likely to be never married and have problems with planning and self-awareness; and were less likely to have indecent contact behavior, indecent exposure, index violent crimes, have a non-violent criminal history, previous sexual offenses, chronicity of sexual offending, coercive sexual behavior, escalation of sexual violence, sexual deviance, and alcohol or substance use problems. The authors concluded that static risk factors were “weak and insufficient” to accurately discriminate offenders with low IQ from those with higher IQ and recommended future studies investigate the dynamic risk factors that appeared to discriminate the two groups.

Finally, Leshem et al. compared the auditory processing of spoken-emotions in 21 men diagnosed with schizophrenia who had been convicted of a violent offense (forensic group) and 45 men with schizophrenia and did not have a violent criminal history (non-forensic group). The authors reported that both groups identified spoken emotions, but the forensic group had better performance in emotion-discrimination than the non-forensic group. They hypothesized that the identification and discrimination of emotions may be affected by schizophrenia to a lesser degree in forensic groups and that the reduced ability to identify emotions in the non-forensic group may induce social withdrawal and reduce the risk of engaging in violence. The authors reported that the two groups did not differ in selective-attention. They recommended that “treatment programs in population must consider symptomatology, traits and history” and that emotion processing “forms a key target in the Integrated Neurocognitive Therapy for schizophrenia patients.”

This Research Topic focused on mental illness within correctional populations. By generating greater understanding of prevalence, risk assessment, and treatment it is the goal for more effective treatment programs to be administered in correctional settings. Innovative programs that deal with core aspects of untreated mental illness, allow a path forward for individuals who otherwise would be at higher risk for reoffending and returning to a correctional facility. This issue is an important, albeit small step in disseminating cutting edge research on mental illness in correctional populations.

## Author contributions

All authors listed have made a substantial, direct, and intellectual contribution to the work and approved it for publication.
